# Association of *GLP1R* locus with mental ill‐health endophenotypes and cardiometabolic traits: A trans‐ancestry study in UK Biobank

**DOI:** 10.1111/dom.16178

**Published:** 2025-01-22

**Authors:** Madeleine M. E. Hayman, Waneisha Jones, Alisha Aman, Joey Ward, Jana Anderson, Donald M. Lyall, Jill P. Pell, Naveed Sattar, Paul Welsh, Rona J. Strawbridge

**Affiliations:** ^1^ School of Cardiovascular and Metabolic Health University of Glasgow Glasgow UK; ^2^ School of Health and Wellbeing University of Glasgow Glasgow UK; ^3^ Deanery of Molecular, Genetic and Population Health Sciences University of Edinburgh Edinburgh UK; ^4^ College of Medical, Veterinary, and Life Sciences, Graduate School University of Glasgow Glasgow UK; ^5^ Cardiovascular Medicine Unit, Department of Medicine Solna Karolinska Institute Stockholm Sweden

**Keywords:** cardiovascular disease, incretin therapy, meta‐analysis, population study, type 2 diabetes

## Abstract

**Aims:**

Glucagon‐like peptide 1 receptor agonists (GLP1RA), used to treat type 2 diabetes and obesity, have been associated with off‐target behavioural effects. We systematically assessed genetic variation in the *GLP1R* locus for impact on mental ill‐health (MIH) and cardiometabolic phenotypes across diverse populations within UK Biobank.

**Materials and Methods:**

All genetic variants with minor allele frequency >1% in the *GLP1R* locus were investigated for associations with MIH phenotypes and cardiometabolic phenotypes. Linear or Logistic regression analyses (adjusted for age, sex, population structure and genotyping chip) were conducted separately in unrelated individuals of self‐reported white British (*N* = 408 774), white European (*N* = 50 314), South Asian (*N* = 7667), multiple‐ancestry groups (*N* = 10 437) or African‐Caribbean (*N* = 7641) subsets. All ancestries were subsequently combined in an inverse variance‐weighted fixed effects meta‐analysis. Bonferroni correction for multiple testing was applied (for number of independent genetic variants).

**Results:**

Associations were identified between *GLP1R* variants and body mass index (BMI), blood pressure and type 2 diabetes in all ancestries. All ancestries except South Asian had significant MIH associations (mood instability: rs111265626‐G, odds ratio [OR] 0.851 [confidence interval, CI 0.79–0.92], risk‐taking behaviour: rs75408972‐T, OR 1.05 [CI 1.03–1.08] or chronic pain: rs9296280‐C, OR 0.645 [CI 0.54–0.78]). The trans‐ancestry meta‐analysis showed mainly consistent effect sizes and directions for metabolic traits, but discordant directions MIH associations. Only signals for chronic pain, stroke and BMI influenced expression of *GLP1R*.

**Conclusions:**

*GLP1R* variants have consistent cardiometabolic effects across ancestries, but effects on MIH phenotypes are more varied. Any observed behavioural changes with GLP1RA are likely not acting directly through *GLP1R*.

## 1 INTRODUCTION

Glucagon‐like peptide‐1 receptor agonists (GLP1RA) have improved the treatment of type 2 diabetes and could revolutionise the treatment of obesity.^1^, ^2^, ^3^ The growing use of GLP1RA incretin mimetics and their long‐term intended use prompt the need to investigate possible side and off‐target effects, and additional impacts.^4^ However, real‐world data on long‐term effects will take decades to collect, so alternative approaches need to be considered.

Mental ill‐health (MIH), comprised of features and diagnoses of mental illness, are frequently comorbid with metabolic diseases such as obesity and type 2 diabetes. Additionally, higher adiposity is a known factor for increased risk for mental illness.^5^, ^6^ Causal studies have shown that depression and obesity have found to bi‐directional associations and/or shared biology.^7^, ^8^ Genetic studies also support the shared biology for MIH and metabolic disease,^9^, ^10^, ^11^ but the mechanisms of these associations remain unclear. Therefore, off‐target MIH impacts of GLP1RA are plausible. Indeed, emerging evidence suggests that GLP1RA may reduce addictive behaviour including smoking and alcohol misuse.^12^, ^13^ Significant smoking cessation has been observed in a pilot study and is currently being investigated.^12^ While these findings are promising, the molecular pathways underlying these behavioural changes remain to be elucidated. Understanding these mechanisms may provide valuable insights into the broader effects of GLP1RA therapy on both metabolic disease and behavioural health.

GLP‐1 receptor agonists reduce body fat^14^ and indirectly lead to reductions in inflammation (as well as improvements in dysglycaemia, neuronal insulin sensitivity and blood pressure) which might mediate some of the mental health impacts observed in this study. Direct effects of GLP1RAs on inflammation have also been proposed, including modulation of immune cells, cytokine production and oxidative stress.^15^ Alternatively, these effects might be attributable to direct effects of altered levels of GLP‐1 receptor.

GLP1RA effects on behaviour prompted analyses of suicide ideation, but no increased risk of suicide was observed.^16^, ^17^, ^18^ Observational and epidemiological studies have shown that there may be neutral^19^ or protective effects^20^ in using this class of drug on MIH symptoms. However, a study based on individuals taking GLP1RA suggests there is increased prescription of anti‐depressants when used for treatment of diabetes.^21^ Awareness of this should be considered as data accumulate. Early evidence in animal models suggest GLP1RA may decrease depressive and anxious symptoms^22^, ^23^ and offer potential new treatment pathways^24^, ^25^; however, comparing these studies to human clinical evidence will not be possible for some time. The value of genetic studies in predicting adverse outcomes of drugs is increasingly recognised.^26^ Few genetic studies exploring the *GLP1R* locus have been published, but most have had limited power (for example *N* = 264) and/or few variants.^27^, ^28^ UK Biobank (UKB) is a population cohort with half a million participants and data for genetic variation, MIH and cardiometabolic traits (CMt). The large sample size and consistent phenotyping enables a systematic assessment of the locus, meaning we are well positioned to investigate potential off‐target effects of perturbing the *GLP1R* signalling pathway.

The aim of this study was to determine if genetic variation in the *GLP1R* locus was associated with MIH and CMt. Including CMt enables exploration of metabolic effects in addition to those reported for obesity and type 2 diabetes and allows for determination of whether any associations were shared or distinct. In addition, we investigated whether there are consistent effects across ancestries using UKB. The study seeks to give insight into *GLP1R* genetic architecture and the potential long‐term implications of GLP1RAs on MIH and cardiometabolic phenotypes. This study would therefore establish a mechanism between GLP1RA and MIH which has yet to be done.

## 2 MATERIALS AND METHODS

### 2.1 *GLP1R* locus

The *GLP1R* locus included the *GLP1R* coding region ±500 kb (Chr 6:39 016 557–39 055 520, GRCh37, https://genome.ucsc.edu/).

### 2.2 UK Biobank

This study used UKB data (project 71932) and was conducted under the generic ethical approval for UKB, granted by the NHS National Research Ethics Service (approval letter dated 29 June 2021, Ref 21/NW/0157).

UKB recruited ~500 000 participants from across the United Kingdom, between 2006 and 2010.^29^, ^30^, ^31^ Participants aged 40–69 years attended one of the 22 assessment centres and completed extensive baseline questionnaires including information on ethnicity, personal and family medical history, medication and lifestyle. Participants underwent a physical examination including blood sampling for genetic analyses. Six to ten years after recruitment, ~150 000 participants were invited to complete an online thoughts and feelings questionnaire.^32^

### 2.3 Phenotypes

MIH and related traits considered in this study were baseline mood instability (#1920, ‘does your mood often go up and down?’; *N*_missing_: 12 672), risk‐taking behaviour (#2040, ‘do you consider yourself to be someone who takes risks?’; *N*_missing_: 18 531) and anhedonia (#2060, ‘over the past two weeks, how often have you had little interest or pleasure in doing things?’ Controls were those who responded, ‘not at all’, with other responses being considered cases; *N*_missing_: 17 886). Neuroticism (#20127) was assessed using the Eysenck Personality Questionnaire (Revised Short Form) which consisted of 12 no/yes (coded 0/1) questions (including #1920), which were summed. Ever smoking considered current smokers and former versus non‐smokers (data field #20116). From the thoughts and feelings questionnaire, self‐reported addiction and probable lifetime generalised anxiety disorder (GAD), bipolar disorder (BD) and major depressive disorder (MDD)^32^ were analysed. The thoughts and feelings questionnaire was designed by a working group of mental health practitioners, to enable assessment of lifetime experience of GAD, BD and MDD based on combinations of variables as well aligned as feasible to those that would be assessed clinically.^32^ Whilst addiction was based on self‐report only, Davis et al.^32^ demonstrated that the frequencies and demographics of the probably lifetime GAD, BD and MDD are comparable with those diagnoses in the UK population.

The baseline CMt assessed were body mass index (BMI, #21001), waist circumference (WC, #48) and waist‐to‐hip ratio (WHR, calculated from waist and hip circumference measurements, #48/#49). Two measures each of systolic and diastolic blood pressure (SBP and DBP, respectively) were recorded, and the average calculated. Ischaemic heart disease (ISH, heart attack/angina; *N*_missing_: 123 134) and stroke were assessed from self‐report of a diagnosis (#6510; *N*_missing_: 137 889). Venous thromboembolism was self‐reported (VTE, deep‐vein thrombosis and/or pulmonary embolism, #6152; *N*_missing_: 155 048). Prior to analyses, SBP and DBP were adjusted for effects of anti‐hypertensive medication where appropriate (SBP_adj_, DBP_adj_, where SBP +15 mmHg and DBP +10 mmHg if using anti‐hypertensive medication^33^). Probable type 2 diabetes was defined as per Eastwood et al. (self‐report of diagnosis and medication use).^34^ Participants responding ‘don't know’ or ‘prefer not to say’ to any question were excluded from analyses (<5%).

### 2.4 Genetic data

The central UKB team extracted DNA from blood samples using standard protocols and conducted genotyping on Affymetrix genotyping chips (Affymetrix UK BiLEVE Axiom or UKB Axiom).^20^ The central UKB team conducted standard genetic quality control on the genotyping data before and after imputation to the Haplotype Reference Consortium and 1000 Genomes phase 3 references panels.^35^

Individuals were categorised into broad ancestry groups, from self‐reported ethnicity data, per Eastwood et al.^34^ For each ancestry group, one of each related pair (second cousins or closer) was excluded. Unrelated individuals were analysed by ancestry group (white British, white European, multiple ancestry, South Asian and African‐Caribbean). The nomenclature was adopted from UKB with the exception ‘multiple ancestry’ which was changed from ‘mixed’ (#2100).

Using PLINK 1.9,^36^ genetic variants within the *GLP1R* locus were selected and filtered for minor allele frequency (MAF) >1% for each ancestry group and the number of independent genetic variants was calculated using PLINK 1.07^37^ (indep‐pairwise function with default settings). Bonferroni correction for the number of independent variants was used to determine significance thresholds: white British, *N*_variants_ = 902, therefore *p* < 5.54 × 10^−5^; European, *N*_variants_ = 880, *p* < 5.68 × 10^−5^; Multiple ancestries, *N*_variants_ = 1123 *p* < 4.45 × 10^−5^; South Asian, *N*_variants_ = 809, *p* < 6.18 × 10^−5^; African‐Caribbean, *N*_variants_ = 2099, *p* < 2.38 × 10^−5^. Of note, multiple testing correction for phenotypes was not performed, because of prior evidence for the role of this locus in cardiometabolic phenotypes, which overlap to a large degree with each other and MIH phenotypes.

### 2.5 Individual ancestry analyses

Individual ancestry analyses were conducted in PLINK 1.9,^36^ using linear or logistic regression assuming an additive genetic model. Analyses were adjusted for age, sex, population structure (genetic principal components 1–8) and genotyping chip, with addition of anti‐hypertensive and lipid‐lowering medication for analyses of ISH and stroke, or BMI and smoking for analyses of VTE.

Conditional analyses were conducted to identify (a) whether multiple signals exist for a single phenotype (for example, analysis of BMI is adjusted for the BMI lead variant) and (b) whether signals for different phenotypes are independent (for example, analysis of BMI is adjusted for the risk‐taking lead variant). Visualisation of linkage disequilibrium (LD) between signals was conducted using Haploview.^38^

### 2.6 Trans‐ancestry meta‐analyses

To assess trans‐ancestry effect consistency, phenotypes with at least one association in any ancestry group were taken forward to meta‐analyses using METAL.^39^ Sensitivity testing was performed, whereby the white British ancestry group was excluded. Significance was defined by Bonferroni correction for the number of independent variants available in ≥4 of 5 ancestry groups (*N*_variants_ = 561, *p* < 8.91 × 10^−5^).

### 2.7 Follow‐up analyses

Lead variants were investigated for genotype‐specific gene expression using the Genotype‐Tissue Expression (GTEx) Portal https://www.gtexportal.org/home/ (accessed April 2024) and variant effect predictor (VEP) was used to assess predicted functional impact of the significant variants in this study (https://grch37.ensembl.org/index.html, accessed April 2024). Co‐localisation (using coloc package, version 5.2.3, in R) was used to assess whether the same phenotype‐associated variants also influenced *GLP1R* gene expression.^40^

## 3 RESULTS

Characteristics of the UKB participants, by ancestry group, are presented in Table 1.

**TABLE 1:** Descriptive table for UK Biobank participants included in this study.

Visit	Phenotypes	White British	White European	Multiple Ancestry	South Asian	African‐Caribbean
Baseline	*N* (% female)	408 774 (54.1)	50 314 (56.2)	10 437 (57.2)	7667 (46.1)	7641 (57.0)
	Age	56.9 (7.99)	55.6 (8.14)	52.4 (8.07)	53.4 (8.46)	51.9 (8.06)
	Lipid‐lowering medication	71 425 (17.6)	8017 (16.1)	1807 (17.8)	2030 (24.4)	1212 (16.3)
	Anti‐hypertensive medication	85 429 (21.0)	9202 (18.5)	2048 (20.2)	2066 (27.9)	7453 (31.4)
	Diastolic blood pressure (adj)	84.4 (11.3)	83.4 (11.3)	83.9 (11.8)	85.3 (11.5)	87.7 (12.4)
	Diastolic Blood pressure (average)	82.3 (10.1)	81.5 (10.2)	81.9 (10.5)	82.5 (10.2)	84.5 (10.7)
	Systolic blood pressure (adj)	141.5 (20.7)	138.4 (20.5)	136.5 (21.0)	139.1 (21.3)	142.5 (21.6)
	Systolic Blood pressure (average)	138.3 (18.6)	135.7 (18.5)	133.5 (18.6)	135.0 (18.6)	137.8 (18.7)
	Body mass index	27.4 (4.76)	27.2 (4.86)	27.0 (4.91)	27.3 (4.46)	29.5 (5.38)
	Waist circumference	90.3 (13.5)	89.6 (13.7)	88.3 (13.0)	91.8 (12.0)	92.9 (12.5)
	Waist‐to‐hip ratio	0.87 (0.09)	0.87 (0.09)	0.87 (0.08)	0.90 (0.09)	0.87 (0.08)
	Ischaemic heart disease	18 732 (6.14)	2128 (5.53)	414 (5.24)	569 (10.2)	264 (5.43)
	Stroke	6308 (2.16)	705 (1.90)	94 (1.24)	127 (2.46)	118 (2.50)
	T2D	17 760 (4.34)	2117 (4.21)	865 (8.29)	1280 (16.7)	812 (10.1)
	VTE	1161 (0.42)	131 (0.38)	16 (0.24)	6 (0.11)	16 (0.32)
	Chronic pain	237 740 (59.2)	29 906 (60.7)	6731 (67.5)	5045 (70.7)	5022 (69.7)
	Ever smoke	185 005 (45.4)	25 676 (51.2)	3990 (38.5)	1597 (21.1)	2283 (30.1)
	Anhedonia	80 150 (20.3)	11 207 (23.2)	3336 (35.5)	2787 (42.9)	2386 (34.4)
	Addiction	7557 (5.78)	1273 (7.72)	166 (7.41)	46 (4.51)	64 (5.82)
	Mood instability	180 660 (45.2)	22 006 (45.0)	4749 (48.7)	3743 (53.1)	3722 (52.2)
	Risk‐taking behaviour	99 966 (25.3)	16 176 (33.7)	3701 (39.2)	2666 (39.5)	2985 (42.1)
	Neuroticism score	4.11 (3.26)	4.15 (3.30)	4.13 (3.39)	4.39 (3.48)	3.69 (3.20)
	Neuroticism score, median (IQR)	4 (1–6)	4 (1–6)	4 (1–7)	4 (1–7)	3 (1–6)
Mental Health Questionnaire	*N* Mental health questionnaire	1,32 107	16 676	2775	1028	1104
	Bipolar disorder	1895 (1.43)	321 (1.92)	51 (2.24)	24 (2.33)	24 (2.17)
	Generalised anxiety disorder	9229 (10.1)	1273 (11.5)	167 (10.9)	73 (10.4)	54 (6.69)
	Major depressive disorder	31 266 (23.67)	4158 (24.93)	538 (39.0)	192 (18.68)	203 (18.39)

*Note*: Binary variables are presented as *N* (%). Continuous variables are presented as mean (SE). Where variables are not normally distributed, they were additional presented as median and IQR.

Abbreviations: IQR, interquartile range; VTE, venous thromboembolism.

MIH in general was common but varied across ancestry groups (Table 1). Due to sample size, analysis for GAD was sufficiently available in only white British, European and multiple ancestries. BD was only analysed in white British and white European ancestry groups. The only disorder with sufficient sample size to analyse across all ancestries was MDD (Table 1).

### 3.1 Individual ancestry analyses demonstrate associations of genetic variants in *GLP1R* locus with MIH and CMt phenotypes

**FIGURE 1:**
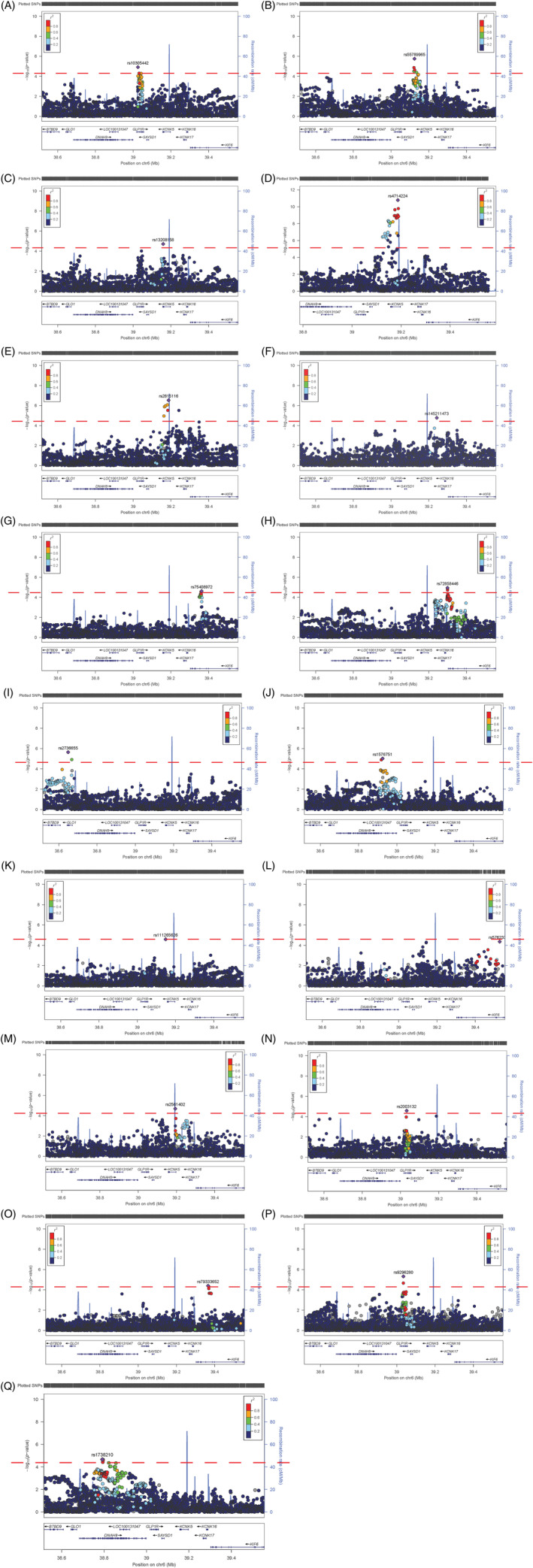
Regional association plots from lead variant analyses, showing where the signal is within the *GL1PR* locus. (A) Body mass index‐associated signal—White British. (B) Type II diabetes (T2D)‐associated signal—White British. (C) Waist circumference (WC)‐associated signal—White British. (D) Diastolic blood pressure (DBP)‐associated signal—White British. (E) Systolic blood pressure (SBP)‐associated signal 1—White British. (F) SBP‐associated signal 2—White British. (G) Risk‐taking behaviour‐associated signal—White British. (H) DBP‐associated signal—White European. (I) T2D‐associated signal—White European. (J) Risk‐taking behaviour‐associated signal—White European. (K) Mood instability‐associated signal—White European. (L) WC‐associated signal—Multiple ancestry. (M) Waist‐to‐hip ratio‐associated signal—Multiple ancestry. (N) Stroke‐associated signal—Multiple ancestry. (O) Generalised anxiety disorder‐associated signal—Multiple ancestry. (P) Chronic pain‐associated signal—African‐Caribbean. (Q) DBP‐associated signal—South Asian.

Across the five ancestry groups, 17 significant genetic associations were observed (Table 2). Upon visual inspection (Figure 1), the signals for the different phenotypes were spread throughout the locus.

**TABLE 2:** Lead signals from single ancestry analyses.

Ancestry	Category	Association	Variant	A1	A2	MAF	OR (beta)	L95/U95 (SE)	*p*‐Value	Type
White British	MIH	Risk‐taking	rs75408972	T	C	0.05	1.05	1.03/1.08	2.37E‐05	Lead
White European	MIH	Risk‐taking	rs1576751	G	A	0.40	0.9391	0.91/0.97	9.65E‐06	Lead
White European	MIH	Mood instability	rs111265626	G	A	0.03	0.8513	0.79/0.92	2.56E‐05	Lead
Multi Ancestry	MIH	GAD	rs79333652	A	C	0.02	6.043	2.56/14.3	4.37E‐05	Lead
African‐Caribbean	MIH/CMT	Chronic pain	rs9296280	C	T	0.04	0.645	0.54/0.78	4.76E‐06	Lead
White British	CMT	DBP	rs4714224	C	G	0.27	(−0.186)	(−0.028)	1.64E‐11	Lead
White European	CMT	DBP	rs72858446	T	C	0.05	(−0.679)	(0.155)	1.12E‐05	Lead
South Asian	CMT	DBP	rs1738210	T	C	0.18	(−1.018)	(0.239)	5.00E‐05	Lead
White British	CMT	SBP	rs2815116	G	A	0.30	(−0.240)	(−0.047)	2.96E‐07	Lead 1
White British	CMT	SBP	rs145211473	G	A	0.02	(−0.643)	(−0.150)	1.68E‐05	Lead 2
White British	CMT	BMI	rs10305442	A	G	0.47	(−0.047)	(−0.011)	1.21E‐05	Lead
White British	CMT	T2D	rs55789965	A	G	0.34	1.06	1.04/1.09	1.75E‐06	Lead
White European	CMT	T2D	rs2736655	A	G	0.15	1.22	1.12/1.32	2.28E‐06	Lead
White British	CMT	WC	rs13208158	A	G	0.02	(−0.429)	(−0.100)	1.93E‐05	Lead
Multi Ancestry	CMT	WC	rs57623330	C	G	0.01	(3.205)	(0.784)	4.37E‐05	Lead
Multi Ancestry	CMT	WHR	rs2561402	C	G	0.05	(−0.009)	(0.002)	2.05E‐05	Lead
Multi Ancestry	CVD	Stroke	rs2003132	A	C	0.38	2.348	1.58/3.49	2.05E‐05	Lead

Abbreviations: BMI, body mass index; CMT, cardiometabolic trait; CVD, cardiovascular disease‐related; DBP, diastolic blood pressure; GAD, generalised anxiety disorder; MAF, minor allele frequency; MIH, mental ill‐health; OR, odds ratio; SBP, systolic blood pressure; SE, standard error; T2D, type II diabetes; WC, waist circumference; WHR, waist‐to‐hip ratio.

In the white British group, genetic variants in the *GLP1R* locus were associated with multiple CMt phenotypes (Table 2 and Figure 1). Specifically, there were significant associations with BMI (rs10305442‐A, Beta −0.047 [standard error, SE −0.011], *p* = 1.21 × 10^−5^) (Figure 1A), type 2 diabetes (rs55789965‐A, odds ratio [OR] 1.06 [95% confidence interval, CI 1.04–1.09], *p* = 1075 × 10^−6^) (Figure 1B), WC (rs13208158‐A, Beta −0.429 [SE −0.100], *p* = 1.93 × 10^−5^) (Figure 1C), DBP_adj_ (rs4714224‐C, Beta −0.186 [SE −0.028], *p* = 1.64 × 10^−11^) (Figure 1D) and SBP_adj_ (rs2815116‐G, Beta −0.240 [SE −0.047], *p* = 2.96 × 10^−7^) (Figure 1E). Conditional analyses exploring possible additional signals demonstrated a second independent association for SBP_adj_ (rs145211473‐G, Beta −0.643 [SE −0.150], *p* = 1.68 × 10^−5^) (Figure 1F) (Table 2, Tables S1–S10). For MIH phenotypes, one significant genetic association was observed with risk‐taking behaviour (rs75408972‐T, OR 1.05 [CI 1.03–1.08], *p* = 2.37 × 10^−5^) (Table 2, Figure 1G, Tables S11 and S12) while a borderline association was observed for anhedonia (rs1005951‐G, OR 0.98 [CI 0.96–0.99], *p* = 5.94 × 10^−5^) (Table S13).

Within the white European group, genetic associations were observed for DBP_adj_ (rs72858446‐T, Beta −0.679 [SE 0.155], *p* = 1.12 × 10^−5^) (Figure 1H) and type 2 diabetes (rs2736655‐A, OR 1.22 [CI 1.12–1.32], *p* = 2.28 × 10^−6^) (Figure 1I) as well as risk‐taking behaviour (rs1576751‐G, OR 0.939 [CI 0.91–0.97], *p* = 9.65 × 10^−6^) (Figure 1J) and association with mood instability (rs111265626‐G, OR 0.851 [CI 0.79–0.92], *p* = 2.56 × 10^−5^) (Table 2, Figure 1K, Tables S14–S21).

In the multiple‐ancestry group, significant genetic associations were observed for WHR (rs2561402‐C, Beta −0.009 [SE 0.002], *p* = 2.05 × 10^−5^) (Figure 1L), WC (rs57623330‐C, Beta 3.205 [SE 0.784], *p* = 4.37 × 10^−5^) (Figure 1M), stroke (rs2003132‐A, OR 2.348 [CI 1.58–3.49], *p* = 2.05 × 10^−5^) (Figure 1N) and GAD (rs79333652‐A, OR 6.043 [CI 2.56–14.3], *p* = 4.37 × 10^−5^) (Table 2, Figure 1O, Tables S22–S29). The high odds ratio for GAD may be due to a small case number and low MAF (167 cases and MAF 0.02).

Within the African‐Caribbean dataset, genetic variants were significantly associated with chronic pain (rs9296280‐C, OR 0.645 [CI 0.54–0.78], *p* = 4.76 × 10^−6^) (Table 2, Figure 1P, Tables S30 and S31). Additionally, a genetic association with mood instability (rs34128177‐C, OR 0.809 [CI 0.73–0.89], *p* = 2.79 × 10^−5^) was borderline significant (Table S32).

In the South Asian ancestry dataset, a genetic association was found with DBP_adj_ (rs1738210‐T, Beta −1.018 [SE 0.239], *p* = 5.00 × 10^−5^) (Table 2, Figure 1Q, Tables S33 and S34). There was also a borderline significant association with ISH (rs2748171‐T, OR 0.619 [CI 0.49–0.78], *p* = 6.37 × 10^−5^) (Table S35).

### 3.2 Trans‐ancestry meta‐analysis shows concordant effects of genetic variation in *GLP1R* on CMt phenotypes but discordant genetic effects on behavioural phenotypes

Results of the significant meta‐analyses are presented in Table 3 (Tables S36–S48). These results demonstrate that BMI was significantly associated with genetic variation in the *GLP1R* locus in all ancestries, and whilst the multiple‐ancestry group has an inconsistent direction, there is no heterogeneity (HetIsq = 0). The type 2 diabetes and SBP signals showed consistent directions in all data subsets and the heterogeneity was very low (HetIsq = 0%–8.9%). For sensitivity analysis, the meta‐analyses were repeated without the white British group, which is the largest group, to determine if this group was driving the association results (Table S50); however, the findings were comparable. Significant associations were identified for risk‐taking behaviour and DBP across all groups; however, the effect direction was inconsistent. The single ancestry lead variants and their results in the trans‐ancestry meta‐analyses are presented in Table S50.

**TABLE 3:** Lead signals from trans‐ancestry meta‐analysis.

Association	Marker name	A1	A2	Freq1	FreqSE	MinFreq	MaxFreq	Effect	SE	*p*‐Value	Direction					HetISq	HetPVal
											WB	WE	MA	SA	AC		
BMI	rs1042044	A	C	0.43	0.01	0.43	0.45	−0.04	0.01	1.95E‐05	−	−	+	−	−	0	0.8418
DBP	rs4714224	C	G	0.27	0.01	0.21	0.28	−0.17	0.03	8.09E‐12	−	−	−	−	+	21.6	0.2768
DBP	rs10947788	T	C	0.28	0.01	0.26	0.29	−0.16	0.03	4.39E‐11	−	−	−	−	+	0	0.7676
DBP	rs72858446	T	C	0.06	0.01	0.02	0.08	−0.22	0.05	5.83E‐06	−	−	−	+	+	68	0.0139
DBP	rs2115200	T	C	0.78	0.01	0.73	0.98	−0.12	0.03	8.18E‐06	−	−	−	−	+	0	0.6268
T2D	rs2490026	T	C	0.47	0.03	0.46	0.62	−0.05	0.01	9.29E‐07	−	−	−	−	−	8.9	0.3557
T2D	rs1781719	t	c	0.49	0.05	0.48	0.76	−0.05	0.01	1.47E‐06	−	−	−	−	−	0	0.6208
Risk‐taking	rs75408972	T	C	0.05	0.00	0.01	0.05	0.05	0.01	5.42E‐06	+	+	+	−	−	0	0.5472
SBP	rs9394578	A	C	0.26	0.01	0.23	0.30	0.26	0.05	8.41E‐09	+	+	+	+	+	0	0.6791

*Note*: The plus (+) indicates increasing direction, the minus (−) indicates decreasing direction.

Abbreviations: AC, African‐Caribbean; BMI, body mass index; DBP, diastolic blood pressure; MA, multiple ancestry; SA, South Asian; SBP, systolic blood pressure; SE, standard error; T2D, type II diabetes; WE, white European; WB, white British.

### 3.3 Assessment of LD between signals

**FIGURE 2:**
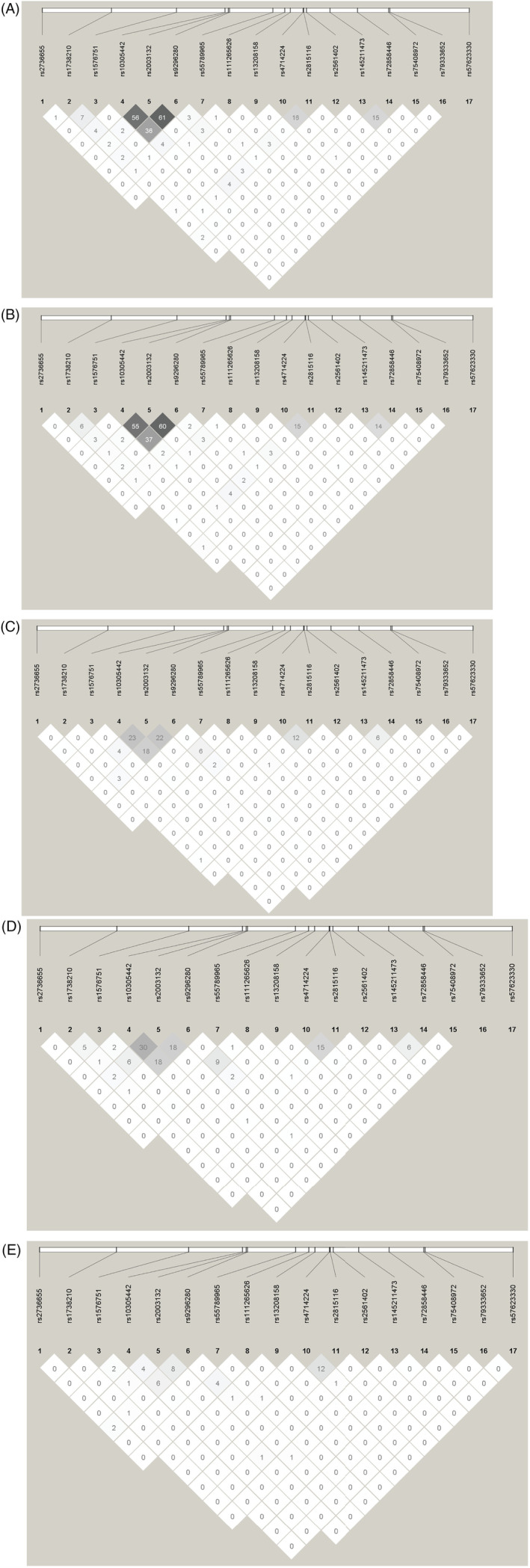
Linkage disequilibrium plots with all significant signals. (A) White British, (B) white European, (C) multiple ancestry, (D) South Asian and (E) African‐Caribbean. Order of signals: 1‐type 2 diabetes—White European, 2‐type 2 diabetes—Meta‐analysis, 3‐type 2 diabetes—Meta‐analysis, 4‐diastolic blood pressure (DBP)—South Asian, 5‐Risk‐taking—White European, 6‐body mass index (BMI)—White British, 7‐Stroke—Multiple ancestry, 8‐Chronic pain—African‐Caribbean, 9‐BMI—Meta‐analysis, 10‐DBP—Meta‐analysis, 11‐type 2 diabetes—White British, 12‐Mood instability—White European, 13‐waist circumference (WC)—White British, 14‐systolic blood pressure (SBP)—Meta‐analysis, 15‐DBP—Meta‐analysis, 16‐DBP—Meta‐analysis, 17‐SBP—White British, 18‐waist‐to‐hip ratio—Multiple ancestry, 19‐SBP—White British, 20‐DBP—Meta‐analysis, 21‐Risk‐taking—Meta‐analysis, 22‐generalised anxiety disorder—Multiple ancestry, 23‐WC—Multiple ancestry.

In the white British dataset (Figure 2A), most variants were independent; however, high LD (defined as *r* ^2^ > 0.8) was observed between the two type 2 diabetes signals, the two DBP signals (meta‐analyses, with 0 and non‐0 HetIsq) and the SBP meta‐analysis and white British leads. Moderate LD (defined as *r* ^2^ > 0.70) was observed between the BMI meta‐analysis and white British leads. Similar patterns of LD were observed in the white European dataset (Figure 2B). In the multiple ancestry (Figure 2C) and South Asian ancestry (Figure 2D) groups, the LD for the type 2 diabetes and SBP signals show a similar pattern, albeit with lower values, but the LD between BMI signals was limited (defined as *r* ^2^ < 0.50). In the African‐Caribbean ancestry group (Figure 2E), there was moderate LD between the SBP meta‐analysis and white British leads, but not the other signals. Overall, these results suggest similarities between the white British analyses and the meta‐analyses (unsurprising, given the white British ancestry same size) and highlight the fact that all other trait signals were independent from each other.

### 3.4 Follow‐up analysis shows that signals demonstrated non‐ *GLP1R* gene expression for MIH endophenotypes

The majority of the lead variants showed no functional significance and were reported as intron variants in VEP (Table S51). The remaining lead variants that had unique functional impacts were rs2748171‐T and rs1042044‐A. The borderline significant ISH‐associated rs2748171‐C was described as a nonsense mediated decay transcript variant for *BTBD9*. The BMI‐ associated rs1042044‐A was reported as a missense variant with moderate impact for *GLP1R* (Table S51). Lead variants demonstrated genotype‐specific effects on expression of nine genes (Table S51). Eleven of the lead variants were not found in GTEx. Of the lead variants from both the lead analysis and meta‐analysis (*N*_signal total_ = 25), three were within the coding region for *GLP1R* (Table S51). Gene expression data within *GLP1R* was associated with the lead variant genotypes for BMI, chronic pain and stroke. No MIH‐associated variants showed genotype‐specific gene expression patterns for *GLP1R* in GTEx. MIH‐associated genotypes for mood instability and risk‐taking behaviour had an association with gene expression levels in two genes: *DNAH8* and *GLO1*. The risk‐taking behaviour‐associated genotype was associated with gene expression data in *KIF6*, *DAAM2* and *BTBD9* (Table S51). Genotype‐specific effects on splicing were observed with the gene expression levels associated with chronic pain, risk‐taking behaviour, SBP and stroke (Table S51). Co‐localisation analyses provided no support for shared causal variants. Only two trait‐associated variants (rs10305442 [BMI, white British] and rs2003132 [stroke, multi ancestry]) had genotype‐specific effects on gene expression and the posterior probability did not support there being a shared causal variant (posterior probabilities 7.13 × 10^−7^ and 6.64 × 10^−7^, respectively, compared to strong evidence of a shared variant where posterior probability ≥0.75).

## 4 DISCUSSION

We report associations of *GLP1R* variants with MIH in white British (risk‐taking behaviour), white European (mood instability and risk‐taking behaviour), multiple ancestry (GAD) and African‐Caribbean (chronic pain) ancestry groups. No MIH associations were found within the South Asian group. We also found associations of *GLP1R* variants with CMt in all ancestry groups. However, there was no evidence to suggest that there were shared effects on CMt and MIH (i.e., the lead variants differ and are in low LD [*r* ^2^ < 0.50]). The meta‐analysis showed consistent effects of *GLP1R* on CMt across ancestries but inconsistent effects on MIH. Seven lead variants were associated with MIH endophenotypes, but none influenced *GLP1R* expression, suggesting that effects of *GLP1R* variants on MIH are likely not acting through *GLP1R*. Consistent effects of *GLP1R* locus variants on CMt were observed across all ancestry groups. There are reports of improvements in lifestyle factors such as reduced smoking; however, clinical assessment of changes in MIH is yet to be adequately assessed to confirm the role that *GLP1R* may play in mental illness.^20^ Our data provide no evidence that these drugs might improve MIH, but more importantly there is no suggestion of worsening of MIH, which supports current literature.^41^ Collectively, these data do not suggest that GLP1RA drugs are likely to induce depression or other serious mental health disorders through the GLP‐1 receptor. However, long‐term randomised controlled trials (supplemented by large‐scale observational data for outcomes of low prevalence) will ultimately be required to assess this conclusively.

Associations with risk‐taking and its significance in the meta‐analysis highlight the potential connection between the *GLP1R* locus and behavioural effects, irrespective of the associated signals being outside of the coding region. There is recent evidence emerging about increased smoking cessation while using GLP1RA. For example, Lengsfeld et al.^5^ described a trial using a GLP1RA (liraglutide), during which individuals were found to abstain from smoking behaviours—and not cravings—but did not show any statistical significance. However, in a pilot randomised controlled trial (RCT) by Yammine et al.,^12^ 21.5% of the treatment group showed smoking cessation when compared to the placebo group. Importantly, while current RCTs are underway to investigate this relationship, there is great interest in what pathways may be involved in this interaction with *GLP1R* and addictive behaviour. It is well established that dopamine pathways are large contributors to addictive behaviours and, in some cases, the development of obesity.^42^ Therefore, it is possible that the association with risk‐taking behaviour may be linked to the positive feedback loop via dopaminergic pathways, as is often present for smokers.^43^ Dopaminergic pathways have also been associated with impulse control pathway often characterised with mechanisms within and around the prefrontal cortex of the brain.^44^, ^45^, ^46^ With the reports of GLP1RA inhibiting smoking behaviour, impulse control is a viable avenue to evaluate the potential mechanisms that may be at work.

The lead for type 2 diabetes (rs2490026‐C associated with decreased risk) in GTEx was observed to have eQTL effects in the putamen, or basal ganglia. These areas of the brain have been implicated in mood disorders such as BD and MDD.^47^, ^48^ We observed genotype‐specific effects on expression of the glyoxalase I (*GLO1*), a protein coding gene that is implicated in inflammation and increased glycation of proteins,^49^ which is important in metabolic processes. While the signals for risk‐taking were not associated with *GLO1*, rs34128177‐A (decreased risk allele associated with mood instability) was associated with increased expression of *GLO1*, and mood instability and risk‐taking behaviour are phenotypically related.^50^, ^51^ It is notable that although we have identified that there is an association with a type 2 diabetes‐associated variant (rs2490026‐C) and a *GLO1* eQTL, it is possible this eQTL affects *GLP1R* given that the same variant was associated with multiple genes.^10^ In considering the associations for risk‐taking behaviour that were found in *KIF6*, it is possible that off‐target effects of GLP1RA could be acting via a neighbouring gene pathway. *KIF6* has been assessed for its involvement in obesity and lipid‐lowering medication response^52^ and with the association to risk‐taking behaviour this connection should be explored further.

The genetic association with GAD in the multiple‐ancestry dataset showed strong effects (OR 6.04 [2.56–14.30]). It should be noted that the prevalence of GAD within the UKB population is low; therefore, this finding requires validation. However, anxiety as a feature may be at higher rates than GAD. The increased risk allele associated with GAD rs7933652‐A (MAF 0.02) was not found in the British, European or South Asian datasets suggesting this may be unique to these populations and should be investigated further to fully understand potential associations with this variant and GAD.

We found no MIH associations within the South Asian ancestry sample; however, the following needs to be considered when interpreting this: the sample size was much smaller than that for the white British sample and under‐reporting of MIH phenotypes in the South Asian community is possible. Indeed, individuals from the South Asian community have the least reported MIH.^53^ Therefore, socio‐cultural aspects and stigma related to poor mental health may be contributors to the null findings. While there were differences in the MIH endophenotypes that were significant across the ancestry groups, the variability in power differences between the datasets is a likely contributor to these significant versus null associations. Further research should look at non‐British/European ancestry cohorts to validate our findings as well as investigating the mechanism and timing of these significant variants across the lifespan.

Limitations of this study include a single assessment of variables that likely vary over time. UKB data, and the genetic signals derived from that data, are restricted to the age groups used within the study from ages 40–69; thus, it possible that at different points in the life cycle these signals may have larger effect sizes such as in early development. In addition, UKB is not representative of the general population, and this has implications for exposure/outcome associations,^54^ so these results require further validation in large cohorts that more closely resemble the United Kingdom and/or global population. The predominant demographic of participants in UKB were individuals of greater socioeconomic status and health; therefore, there is possibility of underestimated effects. Another limitation is the way in which mental health information is collected. In many cases receiving a mental disorder diagnosis is difficult and time consuming, which is why we try to incorporate feature of mental illness to capture individuals who experience MIH but may not have a diagnosis (using the RDoC approach).^55^ Previous studies have shown the utility of single question (e.g., anhedonia and risk‐taking^56^, ^57^) for the investigation of MIH.

We do not know how the signals in this study effect the protein or its function and cannot align these results with drug effects. However, this study provides valuable insights into the genetic architecture of the *GLP1R* locus and its implications for both CMt and MIH endophenotypes. The evidence that *GLP1R* variants influence these traits across different ancestries without directly mediating behavioural changes through *GLP1R* suggests that GLP1RA therapies might be employed broadly in patients with type 2 diabetes and obesity, without undue concern for exacerbating mental health problems. This is particularly reassuring for clinicians who manage treatment regimens for patients facing these complex, comorbid conditions. These data will therefore provide reassurance while further analyses of RCTs and pharmaco‐epidemiological datasets is ongoing.

Our study indicates that behavioural changes associated with GLP1RA therapy may not be directly mediated through the *GLP1R*. This finding is reassuring, suggesting that the mental health effects of these drugs might be minimal. However, it remains important for clinicians to monitor mental health, as they would for any vulnerable patient, during GLP1RA therapy. When considering suicidality, this study does not suggest genetic effects; however, we simply do not have sufficient power to understand any association with suicide ideation. Larger genetic consortia and clinical trials should capture data on suicidal ideation to provide more definitive evidence.

There are multiple pathways through which GLP‐1RAs might directly or indirectly influence mental health, including through body fat,^14^ inflammation^15^ and insulin sensitivity.^58^ Further investigation into these mechanisms is warranted to delineate the pathways through which GLP‐1RAs influence both physical and mental health outcomes.

In summary, this study showed that the *GLP1R* locus was not only associated with CMt phenotypes but also MIH, specifically risk‐taking behaviour, mood instability, chronic paid and GAD. Upon further investigation using GTEx, there was no expression evidence to suggest the variants associated with MIH endophenotypes act via a direct *GLP1R* pathway.

## AUTHOR CONTRIBUTIONS

Rona J. Strawbridge and Paul Welsh conceived and supervised the project. Madeleine M. E. Hayman conducted analyses and drafted the manuscript. Madeleine M. E. Hayman, Paul Welsh and Rona J. Strawbridge interpreted the data. All authors provided critical revision of the manuscript.

## CONFLICT OF INTEREST STATEMENT

Paul Welsh reports grant income from Roche Diagnostics, AstraZeneca, Boehringer Ingelheim and Novartis, outside the submitted work and speaker fees from Novo Nordisk and Raisio outside the submitted work. Naveed Sattar has received grant support paid to his university from AstraZeneca, Boehringer Ingelheim, Novartis and Roche Diagnostics outside the submitted work and has consulted for and/or received speaker honoraria from Afimmune, Amgen, AstraZeneca, Boehringer Ingelheim, Eli Lilly, Hanmi Pharmaceuticals, Janssen, Merck Sharp & Dohme, Novartis, Novo Nordisk, Pfizer, Roche Diagnostics and Sanofi. The other authors have no conflicts of interest to declare.

### PEER REVIEW

The peer review history for this article is available at https://www.webofscience.com/api/gateway/wos/peer-review/10.1111/dom.16178.

## ETHICS STATEMENT

UKB has been granted generic ethical approval by the NHS National Research Ethics Service (approval letter dated 29 June 2021, Ref 21/NW/0157). This study used project number 71932, PI RJ Strawbridge.

## Supporting information


**Data S1.** Supplementary tables.

## Data Availability

UKB data are available to registered researchers via application to the central UKB team https://www.ukbiobank.ac.uk/enable-your-research. Code is available upon request to the corresponding author.
